# Robotic and laparoscopic abdominoperineal resections for anal cancer may improve outcomes compared with open surgery: insights from the National Cancer Database

**DOI:** 10.1007/s00464-026-12925-z

**Published:** 2026-06-02

**Authors:** Kayvan Barekatain, Emily F. Simon, Shari Tian, Jay Han, Marnie L. Abeshouse, Kristen M. Westfall, Emily Steinhagen, Meagan Costedio, Ronald A. Charles

**Affiliations:** 1 Department of Surgery, University Hospitals Cleveland Medical Center, 11100 Euclid Ave, Cleveland, OH 44106 USA; 2 Case Western Reserve University School of Medicine, Cleveland, OH USA

**Keywords:** Abdominoperineal resection, Anal cancer, Robotic surgery, Minimally invasive surgery, Laparoscopic surgery, Surgical outcomes

## Abstract

**Background:**

Abdominoperineal resection (APR) is an important treatment option for anal cancer patients with incomplete response or recurrence after chemoradiation, yet the frequency and effectiveness of minimally invasive surgery (MIS) remain poorly described. This study examines trends and outcomes of robotic, laparoscopic, and open APR using the National Cancer Database (NCDB).

**Methods:**

Patients undergoing APR for anal cancer in the NCDB (2010–2022) were categorized by surgical approach based on the initial operative technique, approximating an intention-to-treat framework. The study period was divided into early (2010–2016) and late (2017–2022) eras to assess temporal trends. Demographics, tumor characteristics, and perioperative outcomes were compared. Primary endpoints included 30- and 90-day mortality, length of stay, margin positivity, and overall survival.

**Results:**

Among 3756 patients (831 robotic, 723 laparoscopic, 2202 open), baseline characteristics were similar across approaches. Robotic APR increased significantly in the late era (33.3% vs 13.2%, *p* < 0.001), whereas open APR declined (48.2% vs 66.9%) and laparoscopic APR remained stable (18.5% vs 19.9%). MIS approaches demonstrated lower 30-day mortality (1.2% robotic vs 1.7% laparoscopic vs 2.2% open, *p* < 0.001), lower 90-day mortality (3.2% vs 3.6% vs 4.9%, *p* < 0.001), and fewer margin positivity rates (14.4% vs 14.1% vs 17.8%; *p* < 0.001). Median length of stay was 5, 6, and 8 days for robotic, laparoscopic, and open, respectively (*p* < 0.001). Five-year overall survival was 56%, 54%, and 49%. On Cox regression adjusted for age, year, comorbidity, and stage, open APR was associated with a 25% higher hazard of all-cause mortality compared with robotic APR (HR 1.247, CI 1.061–1.466, *p* = 0.007), while laparoscopic and robotic demonstrated comparable survival.

**Conclusions:**

Minimally invasive APR for anal cancer has increased over time and is associated with shorter hospital stays, lower perioperative mortality, fewer positive margins, and improved survival. These findings support laparoscopic and robotic APR as preferred approaches for appropriately selected anal cancer patients.

Anal squamous cell carcinoma is a rare malignancy accounting for approximately 3.0% of gastrointestinal cancers in the United States, with an estimated 10,930 new cases and 2030 deaths in 2025 [1]. The incidence of anal cancer has been rising globally, with age-adjusted death rates increasing by an average of 5.1% annually from 2013 to 2022 [2]. Most cases are linked to human papillomavirus infection, with additional risk factors including HIV infection and smoking [3].

Before the 1970s, anal cancer was managed with radical abdominoperineal resection (APR) and permanent colostomy, resulting in 5-year overall survival rates of 40–70% and locoregional recurrence rates as high as 50% [4]. In 1974, the introduction of the Nigro protocol, consisting of concurrent chemoradiation with fluorouracil and mitomycin-C, established a new treatment paradigm [4]. The excellent outcomes from this approach, including 70–90% survival and avoidance of surgical morbidity and colostomy, have made this treatment paradigm the standard of care [3].

Despite these advances, approximately 20–30% of patients experience incomplete responses or recurrence after chemoradiation [4]. For these patients, abdominoperineal resection remains the primary salvage treatment option. According to NCCN guidelines, APR is indicated for local recurrence following a complete response or for persistent or progressive disease after definitive chemoradiation [3]. Salvage APR is associated with 5-year overall survival rates of 25–50% and involves substantial morbidity, particularly delayed perineal wound healing in about two-thirds of patients [5].

The surgical approach to pelvic malignancies has significantly evolved over the past two decades, with minimally invasive surgery (MIS) techniques increasingly replacing open procedures. For colorectal cancer, high-quality evidence shows that laparoscopic approaches achieve comparable oncologic outcomes to open resection, while offering perioperative advantages such as reduced blood loss, quicker return of bowel function, shorter hospital stays, and faster recovery [6]. Population-based studies also corroborate these advantages, indicating that minimally invasive surgery is associated with lower rates of adhesive small bowel obstruction and surgical site infections, and shorter hospital stays [7, 8].

More recently, robotic surgery has emerged as an alternative to a laparoscopic approach, particularly for low rectal and anal cancers where the confined pelvic space presents technical challenges for conventional laparoscopy. The robotic platform offers three-dimensional visualization, a stable camera platform, and flexible robotic arms with wristed instruments that can move freely in narrow spaces, potentially overcoming limitations of laparoscopic surgery [5]. The adoption of robotic surgery for rectal cancer has increased exponentially, with utilization rising from approximately 4% in 2010 to 34% by 2019, surpassing laparoscopic approaches in some centers [6].

The recent REAL randomized clinical trial demonstrates that robotic surgery achieves lower locoregional recurrence and improved disease-free survival compared with laparoscopic approaches for rectal cancer [5]. For APR specifically, robotic surgery has been associated with lower conversion rates, fewer postoperative complications, reduced margin positivity, and shorter hospital stays compared with both laparoscopic and open approaches [7].

Despite this growing evidence supporting minimally invasive approaches for rectal cancer, there is a dearth of large-scale studies addressing the oncologic equivalence and perioperative benefits of minimally invasive APR in the anal cancer population. Anal cancer patients often undergo prior pelvic radiation with wider treatment fields that include the inguinal nodes, resulting in significant tissue fibrosis and the need for wider perineal margins compared with rectal cancer surgery [3]. These differences are partially reflected in the substantially higher rates of myocutaneous flap reconstruction required for perineal closure in anal cancer compared with rectal cancer patients undergoing APR [8].

This study fills a knowledge gap by analyzing trends and outcomes of robotic, laparoscopic, and open abdominoperineal resection for anal cancer using the National Cancer Database from 2010 to 2022. We hypothesized that minimally invasive approaches would show perioperative benefits and be oncologically equivalent to open surgery, similar to findings in rectal cancer populations. Understanding the relative effectiveness of these surgical approaches is essential for optimizing treatment strategies and improving outcomes for anal cancer patients who need salvage surgery after chemoradiation.

## Materials and methods

### Study design and data source

A retrospective cohort study was performed using the National Cancer Database (NCDB). The NCDB is a nationwide, hospital-based oncology registry jointly sponsored by the American College of Surgeons Commission on Cancer and the American Cancer Society, capturing approximately 70% of newly diagnosed malignancies in the United States from Commission on Cancer-accredited centers. The dataset is de-identified; therefore, this study was exempt from Institutional Review Board review.

### Inclusion and exclusion

Patients were included if they were diagnosed with anal cancer between 2010 and 2022 and underwent surgical treatment with abdominoperineal resection (APR). APR was defined by the following variables in the NCDB (rx_summ_surg_prim_site codes 60–63). Patients were excluded if they underwent no surgery, local destruction, local excision, surgery not otherwise specified, or had unknown surgery status. Patients without a coded surgical approach were excluded. All patients were aged 18 or older, and patients aged 90 or older were coded as 90 years of age as predefined in the NCDB. Clinical stage group (Stage 0–IV) was derived from clinical TNM staging variables, incorporating both legacy (pre-2018) and updated AJCC coding.

### Demographics

Other demographic variables included sex (defined as binary male/female), race (defined as non-Hispanic White, non-Hispanic Black, Hispanic, and Other), Charlson–Deyo Comorbidity Score (defined as an ordinal variable ranging from 0 to 3 or more, with 0 representing no comorbidities), patient income (defined as median income quartiles in quartiles), insurance status (defined as uninsured, private, Medicaid or Medicare, Other government, and unknown), and treatment facility (defined as community cancer program, comprehensive community cancer program, academic/research program, integrated network cancer program, and unknown). The year of diagnosis was coded as a categorical variable: those diagnosed from 2010 to 2016 and those from 2017 to 2022.

### Exposure

The primary exposure was the surgical approach, identified using the NCDB surgical approach variable (rx_hosp_surg_appr_2010). The surgical approach was analyzed using an intention-to-treat framework and categorized as follows: robotic (robotic and robotic converted to open), laparoscopic (laparoscopic and laparoscopic converted to open), and open (open).

### Outcomes

Primary perioperative outcomes included 30-day mortality, 90-day mortality, unplanned 30-day readmission, length of stay, and margin positivity (R0 vs R+). The long-term outcome was overall survival, defined as time from diagnosis to death or last follow-up.

Mortality variables in the NCDB include an “unknown” category reflecting missing status; mortality comparisons therefore reflect differences in the combined distribution of alive/dead/unknown across groups. Mortality rates among patients with known status are described in the Results.

#### Statistical analysis

Analyses were performed in StataSE v 17.0 (StataCorp, College Station, TX). Baseline demographics, tumor characteristics, and perioperative outcomes were compared across surgical approaches. Continuous variables are reported as median (IQR) and compared using Kruskal–Wallis tests. Categorical variables are summarized as frequency (%) and compared using chi-square tests. Multivariable logistic regression adjusting for clinical stage was performed to evaluate the independent association between surgical approach and margin positivity. Interaction between surgical approach and stage was tested using multiplicative interaction terms.

Overall survival (OS), measured in months from NCDB date of diagnosis to death or last contact, was evaluated with Cox proportional hazards models to estimate adjusted hazard ratios (HRs). Covariates included age, sex, race/ethnicity, year of diagnosis, Charlson–Deyo comorbidity score, and clinical stage group. Patients with missing covariate data were excluded from multivariable analyses through complete-case methodology. Hazard ratios (HR) with 95% confidence intervals (CI) are reported. Statistical significance was defined as a two-sided *p*-value < 0.05.

## Results

### Patient and tumor characteristics

**Fig. 1:**
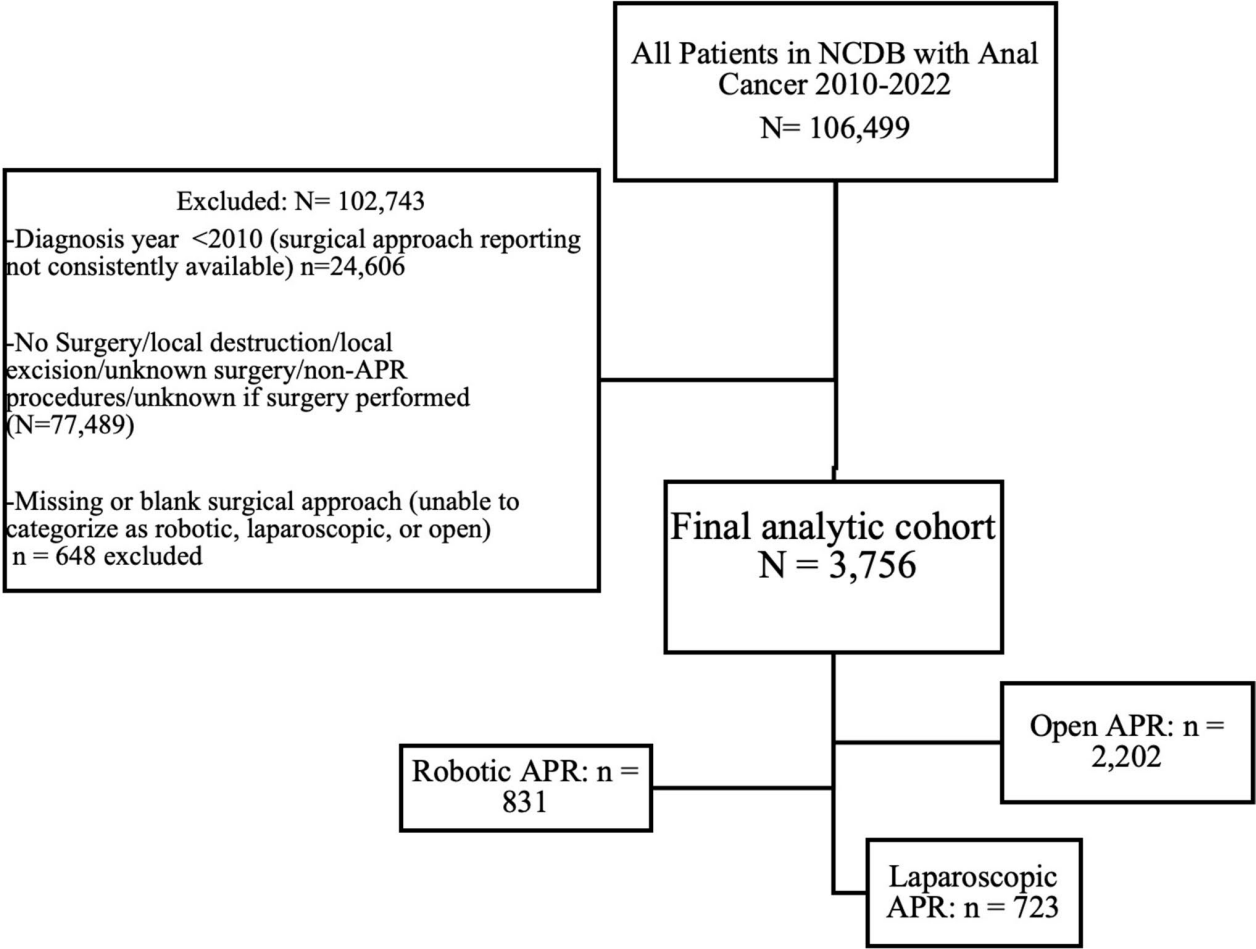
Study cohort selection flow diagram

A total of 106,499 patients diagnosed with anal cancer between 2010 and 2022 were available in the NCDB. After applying inclusion and exclusion criteria, 3756 patients underwent APR for anal cancer between 2010 and 2022, including 831 robotic, 723 laparoscopic, and 2202 open resections (Fig. 1). Roughly 50% (*n* = 1877) of patients were male, 77% were non-Hispanic White (*n* = 2879), and the median patient age was 63 years old. Most patients had low Charlson–Deyo Comorbidity (CDC) scores, with approximately 90% having scores less than or equal to 1. Age, sex distribution, race/ethnicity, and Charlson–Deyo Comorbidity (CDC) scores were comparable across surgical approaches (all *p* > 0.05).

Income quartiles differed significantly across groups (*p* = 0.030), with open APR more frequently performed among lower-income quartiles. Facility types also differed (*p* = 0.039) with robotic and laparoscopic approaches more commonly performed at academic/research programs.

Among patients with available clinical stage group data, stage distribution was similar across approaches (*p* = 0.18). Stage I–III disease accounted for approximately 90% of cases across all surgical approaches, and stage IV disease was present in 5–6% of patients in each group. Clinical stage group data were missing in 30–37% of patients across surgical approach groups. For multivariable analyses, clinical stage was included as a covariate, and patients with missing stage were excluded from adjusted Cox regression models. Further demographic information can be viewed in Table 1.

**Table 1:** Demographic and staging of patients by surgical approach

Demographics	Robotic *N* = 831	Laparoscopic *N* = 723	Open *N* = 2202	*p*-value
Age	64 (56–72)	63 (54–73)	63 (54–72)	0.091
Year of diagnosis				
2010–2016	276	415	1399	< 0.001
2017–2022	555	308	803	
Sex				
Male	440 (52.9%)	365 (50.5%)	1072 (48.7%)	0.11
Female	391 (47.1%)	358 (49.5%)	1130 (51.3%)	
Race				
Non-Hispanic White	651 (78.3%)	549 (75.9%)	1679 (76.2%)	0.86
Non-Hispanic Black	97 (11.7%)	98 (13.6%)	298 (13.5%)	
Hispanic	41 (4.9%)	40 (5.5%)	110 (5.0%)	
Other	42 (5.1%)	36 (5.0%)	115 (5.2%)	
Charlson–Deyo score				
0	594 (71.5%)	532 (73.6%)	1591 (72.3%)	0.47
1	154 (18.5%)	123 (17.0%)	360 (16.3%)	
2	46 (5.5%)	35 (4.8%)	122 (5.5%)	
3 or more	37 (4.5%)	33 (4.6%)	129 (5.9%)	
Median income				
< $46,277	123 (14.8%)	111 (15.4%)	383 (17.4%)	0.030
$46,277–$57,856	185 (22.3%)	132 (18.3%)	438 (19.9%)	
$57,857–$74,062	174 (20.9%)	128 (17.7%)	441 (20.0%)	
$74,063+	245 (29.5%)	227 (31.4%)	613 (27.8%)	
Unknown	104 (12.5%)	125 (17.3%)	327 (14.9%)	
Insurance status				
Uninsured	22 (2.6%)	24 (3.3%)	75 (3.4%)	0.50
Private	294 (35.4%)	254 (35.1%)	772 (35.1%)	
Medicaid or Medicare	494 (59.4%)	424 (58.6%)	1299 (59.0%)	
Other government	16 (1.9%)	11 (1.5%)	25 (1.1%)	
Unknown	27 (3.2%)	16 (2.2%)	73 (3.3%)	
Facility type				
Community cancer program	21 (2.5%)	30 (4.1%)	85 (3.9%)	0.039
Comprehensive community cancer program	284 (34.2%)	240 (33.2%)	669 (30.4%)	
Academic/research program	335 (40.3%)	313 (43.3%)	1005 (45.6%)	
Integrated network cancer program	164 (19.7%)	124 (17.2%	370 (16.8%)	
Unknown	27 (3.2%)	16 (2.2%)	73 (3.3%)	
TNM staging				
Stage 0	9 (1.6%)	15 (3.1%)	41 (3.0%)	0.18
Stage 1	209 (36.1%)	176 (36.0%)	465 (33.5%)	
Stage 2	123 (21.2%)	127 (26.0%)	354 (25.5%)	
Stage 3	206 (35.6%)	146 (29.9%)	443 (32.0%)	
Stage 4	32 (5.5%)	25 (5.1%)	83 (6.0%)	

### Temporal trends

The use of robotic APR increased substantially over time. In the early era (2010–2016), 276 of 2090 APRs (13.2%) were performed robotically, compared with 555 of 1666 APRs (33.3%) in the late era (2017–2022) (*p* < 0.001). Although overall APR volume in these groups declined by approximately 20% between eras, the proportional use of robotics more than doubled. During the same period, open APR decreased from 66.9 to 48.2%, whereas laparoscopic utilization remained relatively stable (19.9% vs 18.5%). These findings suggest that increased robotic adoption coincided with a decline in open APR, while laparoscopic utilization remained relatively stable (Table 2).

**Table 2:** Perioperative outcomes by surgical approach

Perioperative outcome	Robotic *N* = 831	Laparoscopic *N* = 723	Open *N* = 2202	*p*-value
Length of stay (days)	5 (4–8)	6 (4–9)	8 (5–12)	< 0.001
Readmission				
No unplanned readmission	752 (90.5%)	661 (91.4%)	2034 (92.4%)	0.15
Unplanned readmission	75 (9.0%)	54 (7.5%)	157 (7.1%)	
Unknown	4 (0.5%)	8 (1.1%)	11 (0.5%)	
Surgical margin				
Negative (R0)	707 (85.6%)	604 (85.9%)	1772 (82.2%)	0.016
Positive (R+)	119 (14.4%)	99 (14.1%)	384 (17.8%)	
30-day mortality				
Alive	731 (88.8%)	678 (94.6%)	2061 (94.0%)	< 0.001
Dead	9 (1.1%)	12 (1.7%)	46 (2.1%)	
Unknown	83 (10.1%)	27 (3.8%)	85 (3.9%)	
90-day mortality				
Alive	708 (86.7%)	661 (92.6%)	1991 (91.2%)	< 0.001
Dead	26 (3.2%)	26 (3.6%)	106 (4.9%)	
Unknown	83 (10.2%)	27 (3.8%)	85 (3.9%)	

### Perioperative outcomes

Perioperative outcomes differed significantly by approach. Median length of stay was shortest among robotic cases at 5 days (IQR 4–8), compared with 6 days (IQR 4–9) for laparoscopic and 8 days (IQR 5–12) for open APR (*p* < 0.001). Rates of unplanned readmission were similar across groups (robotic 9.0%, laparoscopic 7.5%, open 7.1%; *p* = 0.15).

Margin positivity differed by surgical approach, occurring in 14.4% of robotic cases, 14.1% of laparoscopic cases, and 17.8% of open cases (*p* = 0.016). Margin positivity increased with advancing stage (*p* < 0.001). In multivariable logistic regression adjusting for clinical stage, open surgery remained independently associated with higher odds of positive margins compared with robotic surgery (OR 1.43, *p* = 0.013), whereas laparoscopic surgery was not significantly different from robotic. No significant interaction was observed between surgical approach and clinical stage (*p* = 0.96).

Thirty- and ninety-day mortality differed across surgical approaches (both *p* < 0.001). However, mortality status was more frequently missing among robotic cases (30-day mortality unknown: 10.1% robotic vs 3.8% laparoscopic vs 3.9% open; 90-day mortality unknown: 10.2% robotic vs 3.8% laparoscopic vs 3.9% open). Given this differential missingness, perioperative mortality comparisons across approaches should be interpreted with caution. Among patients with known mortality status, 30-day mortality was low for all approaches: 1.2% for robotic, 1.7% for laparoscopic, and 2.2% for open APR, while 90-day mortality was 3.2%, 3.6%, and 4.9%, respectively, though these rates may not fully represent true mortality, particularly in the robotic cohort.

### Overall survival

**Fig. 2:**
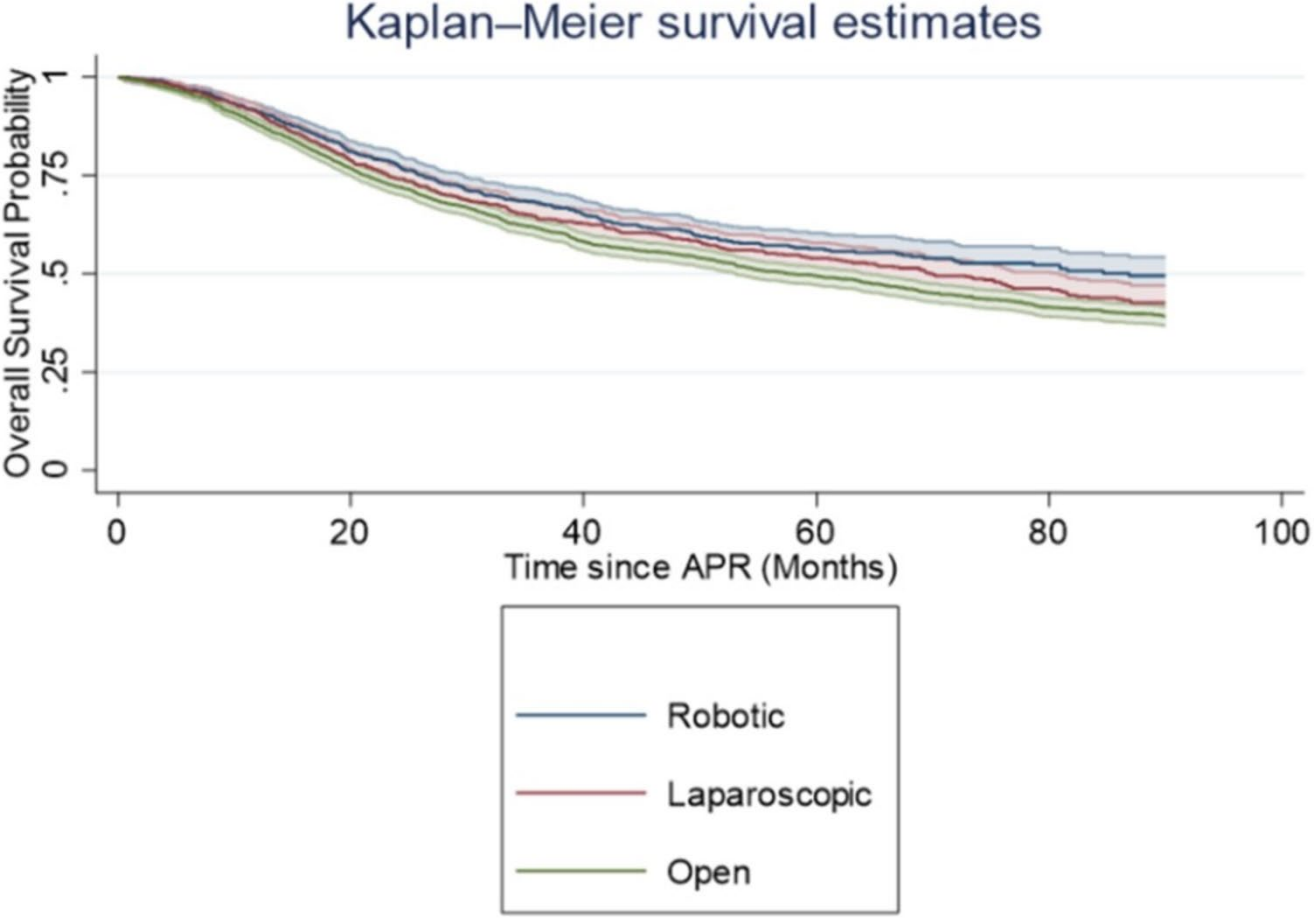
Kaplan–Meier survival estimates by surgical approach

Overall survival measured from date of diagnosis differed significantly by surgical approach (log-rank *χ*^2^ = 14.3, *p* = 0.0008). Kaplan–Meier survival estimates at 6, 12, 24, 36, 48, and 60 months demonstrated consistently higher survival probabilities in the minimally invasive groups compared with open APR (Fig. 2). At 60 months, overall survival was 0.563 (95% CI 0.521–0.603) for robotic, 0.540 (95% CI 0.499–0.578) for laparoscopic, and 0.495 (95% CI 0.472–0.517) for open APR. The similar survival observed between robotic and laparoscopic approaches suggests that the benefit may be attributed to minimally invasive technique broadly rather than the robotic platform specifically.

### Multivariable Cox regression

Multivariable Cox regression results are presented in Table 3. After adjustment for age, diagnosis era, sex, race/ethnicity, comorbidity score, median income, insurance status, facility type, and clinical stage group, open APR remained independently associated with worse overall survival compared with robotic APR (HR 1.247, 95% CI 1.061–1.466, *p* = 0.007). Survival after laparoscopic APR did not differ significantly from robotic APR (HR 1.152, 95% CI 0.949–1.400, *p* = 0.153).

**Table 3:** Adjusted Cox regression demonstrating hazard ratio for overall survival

	Hazard ratio	*p*-value	Confidence interval	
Surgical approach				
Robotic	Reference			
Laparoscopic	1.152	0.153	0.949	1.400
Open	1.247	0.007	1.061	1.466
Age	1.020	0.000	1.014	1.026
Year of diagnosis				
2010–2016	Reference			
2017–2022	0.970	0.650	0.850	1.110
Sex				
Male	Reference			
Female	0.866	0.017	0.770	0.974
Race				
Non-Hispanic White	Reference			
Non-Hispanic Black	0.910	0.312	0.757	1.093
Hispanic	0.730	0.052	0.532	1.003
Other	1.020	0.888	0.778	1.340
Charlson–Deyo score				
0	Reference			
1	1.096	0.241	0.940	1.278
2	1.496	0.002	1.159	1.930
3 or more	1.626	0.000	1.260	2.099
Median income				
< $46,277	Reference			
$46,277–$57,856	0.894	0.258	0.735	1.086
$57,857–$74,062	0.793	0.023	0.650	0.968
$74,063+	0.737	0.002	0.610	0.890
Unknown	0.926	0.469	0.752	1.140
Insurance status				
Uninsured	Reference			
Private	0.911	0.591	0.657	1.281
Medicaid or Medicare	1.142	0.444	0.812	1.607
Other government	1.310	0.396	0.702	2.441
Unknown	1.173	0.570	0.662	2.111
Facility type				
Community cancer program	Reference			
Comprehensive community cancer program	1.051	0.757	0.767	1.440
Academic/research program	0.912	0.561	0.668	1.302
Integrated network cancer program	0.937	0.697	0.674	1.302
Unknown	1.315	0.290	0.792	2.183
TNM staging				
Stage 0	Reference			
Stage 1	1.571	0.070	0.964	2.560
Stage 2	1.921	0.009	1.176	3.140
Stage 3	2.081	0.003	1.278	3.389
Stage 4	4.622	0.000	2.752	7.764

Increasing age was independently associated with mortality risk (HR 1.020 per year, *p* < 0.001) corresponding to a 22% higher hazard per decade of age. Female sex was associated with improved survival compared with male sex (HR 0.866, 95% CI 0.770–0.974, *p* = 0.017). Higher comorbidity burden significantly increased mortality risk (Charlson–Deyo 2: HR 1.496, *p* = 0.002; Charlson–Deyo ≥ 3: HR 1.626, *p* = 0.000). Higher income was associated with survival, with highest quartile earners demonstrating largest improvement (HR 0.737, *p* = 0.002). Clinical stage was strongly associated with survival, with Stage IV disease demonstrating markedly worse survival compared with Stage 0 (HR 4.62, *p* = 0.000).

## Discussion

In this national cohort of 3756 patients undergoing APR for anal cancer, we saw a significant shift toward robotic surgery, with robotic use more than doubling while open APR dropped by nearly 20 percentage points. Laparoscopic procedures remained steady, indicating that robotic adopters probably came from the open surgery group. This shift was linked to better perioperative outcomes, lower margin positivity rates, and improved overall survival with minimally invasive approaches, suggesting that the technical benefits seen in rectal cancer surgery might also apply to the unique challenges of salvage APR in anal cancer patients.

The survival benefit linked to minimally invasive surgery requires careful interpretation. After adjusting for age, comorbidity, clinical stage, and diagnosis era, open APR was associated with a 25% higher hazard of death compared to robotic APR. This link remained even after controlling for known prognostic factors, indicating that the surgical approach may be a modifiable factor influencing outcomes rather than just a marker of patient selection. However, the NCDB does not include variables that could influence both surgical approach and outcomes, such as tumor fixation, extent of local invasion, the expected need for myocutaneous flap reconstruction, and surgeon or institutional expertise. These factors mean the surgical approach may be a poor substitute for these variables. The similar survival rates seen with laparoscopic and robotic approaches support the idea that the benefits are due to minimally invasive techniques overall rather than robotic technology alone, although the robotic platform might offer ergonomic and technical advantages that help with adoption and performance, especially in narrow pelvic spaces.

The lower margin positivity rates observed with minimally invasive approaches (14.4% robotic, 14.1% laparoscopic vs 17.8% open) are particularly clinically relevant given the prognostic importance of R0 resection in salvage APR. Multiple institutional series have demonstrated that margin status is among the strongest predictors of survival following salvage surgery. A 30-year single-institution experience found that radical resection was significantly associated with impaired overall survival [9]. A recent prospective cohort also found that R0 resection was achieved in 80.8% of salvage APR cases and was independently associated with improved disease-free survival (HR 0.24) [10]. The improved visualization and instrument articulation afforded by minimally invasive platforms may facilitate more precise dissection in the fibrotic, previously irradiated tissue planes encountered during salvage APR, potentially explaining the observed differences in margin rates. However, the NCDB does not capture surgical intent, and it is possible that more technically challenging cases, including those with extensive local invasion or anticipated palliative resections, were preferentially managed with open surgery, which would potentially bias comparisons in favor of minimally invasive approaches.

The temporal trends in our study parallel the broader adoption of minimally invasive surgery for pelvic malignancies documented in rectal cancer literature. The approximately 20% decline in overall APR volume between eras may reflect improved chemoradiation efficacy and patient selection. Notably, while the REAL trial and other rectal cancer studies have established the benefits of robotic surgery for primary resection, our findings suggest these advantages translate to the salvage setting despite the added technical complexity of operating in irradiated tissue. The 2025 meta-analysis comparing robotic versus laparoscopic APR for rectal cancer found similar benefits in conversion rates and margin positivity, and our data extend this evidence to a population where such technical advantages may be even more consequential given the hostile operative field [7].

It is possible that some of the differences observed between surgical approaches reflect patient selection rather than technique alone. Patients managed with open surgery were more likely to be treated at non-academic centers and to reside in lower-income areas. These patterns align with the broader literature documenting disparities in access to minimally invasive surgical expertise [11]. However, the persistence of the survival association after multivariable adjustment, combined with biologically plausible mechanisms of reduced surgical trauma and improved margin clearance, supports the interpretation that minimally invasive technique contributes independently to outcomes.

This study is constrained by several limitations inherent to the NCDB. First, the database captures approximately 70% of newly diagnosed cancers from Commission on Cancer-accredited centers, which may limit generalizability to community practice settings where minimally invasive expertise may be less available. Second, the NCDB does not capture cancer-specific survival or disease recurrence, precluding analysis of these important oncologic endpoints that are particularly relevant in salvage surgery where local control is a primary goal. Third, mortality status was more frequently missing among robotic cases (10.1% vs 3.8–3.9%), potentially reflecting the more recent adoption of robotic surgery and shorter follow-up in this cohort; this differential missingness may introduce bias in mortality comparisons. Fourth, we cannot determine whether patients underwent primary APR or salvage APR after failed chemoradiation, which represent distinct clinical scenarios with different expected outcomes and technical challenges. The NCDB also lacks granular data on surgeon experience, case volume, and specific indications for the surgical approach, each of which may independently influence outcomes. Finally, as with all retrospective database studies, residual confounding from unmeasured variables cannot be excluded. Open surgery may be preferentially selected for more complex or advanced salvage cases, which inherently carry worse prognoses. As a result, the observed survival differences between approaches may partially reflect case selection rather than the surgical technique itself, and these associations should not be interpreted as establishing causality.

Our findings have practical implications for surgical planning in anal cancer. The observed benefits of minimally invasive APR support consideration of laparoscopic or robotic approaches for appropriately selected patients, though the decision should be individualized based on tumor characteristics, surgeon expertise, and institutional resources. The concentration of minimally invasive surgery at academic centers and among higher-income populations raises important questions about equitable access; efforts to expand training and understand barriers to adoption in community settings may help ensure these benefits reach all patients requiring salvage APR. In conclusion, robotic and laparoscopic approaches for abdominoperineal resection in anal cancer have increased over time, mirroring trends seen in rectal cancer. Minimally invasive techniques are associated with shorter hospital stays, lower perioperative mortality, fewer positive margins, and improved long-term survival. These findings support laparoscopic and robotic APR as preferred approaches for appropriately selected anal cancer patients who require salvage surgery after chemoradiation.
